# Molecular characterization of *Dictyocaulus* nematodes in wild red deer *Cervus elaphus* in two areas of the Italian Alps

**DOI:** 10.1007/s00436-022-07773-4

**Published:** 2023-01-14

**Authors:** Alessandra Cafiso, Michele Castelli, Perla Tedesco, Giovanni Poglayen, Clelia Buccheri Pederzoli, Serena Robetto, Riccardo Orusa, Luca Corlatti, Chiara Bazzocchi, Camilla Luzzago

**Affiliations:** 1 Department of Veterinary Medicine and Animal Sciences, University of Milan, Lodi, LO Italy; 2 Department of Biology and Biotechnology, University of Pavia, Pavia, PV Italy; 3 Department of Veterinary Medical Sciences, University of Bologna, Ozzano dell’Emilia, BO Italy; 4 Istituto Zooprofilattico Sperimentale Piemonte, Liguria e Valle d’Aosta, National Reference Centre for Wildlife Disease (CeRMAS), Quart, AO Italy; 5 Stelvio National Park, Bormio, SO Italy; 6 Wildlife Ecology and Management, University of Freiburg, Freiburg, Germany; 7 Coordinated Research Center “EpiSoMI”, Università Degli Studi Di Milano, Milan, Italy

**Keywords:** *Dictyocaulus*, Red deer, Molecular analyses, *coxI*, Italy, Geographical clustering

## Abstract

Nematodes of the genus *Dictyocaulus* are the causative agents of parasitic bronchitis and pneumonia in several domestic and wild ungulates. Various species have been described in wild cervids, as the case of *Dictyocaulus cervi* in red deer, recently described as a separate species from *Dictyocaulus eckerti*. In Italy, information on dictyocaulosis in wildlife is limited and often outdated. In this work, 250 red deer were examined for the presence of *Dictyocaulus* spp. in two areas of the Italian Alps (*n* = 104 from Valle d’Aosta, *n* = 146 from Stelvio National Park), and the retrieved lungworms were molecularly characterized. Lungworms were identified in 23 and 32 animals from Valle d’Aosta and Stelvio National Park, respectively. The nematodes, morphologically identified as *D. cervi*, were characterized molecularly (18S rDNA, *ITS2*, and *coxI*). Consistently, almost all specimens were found to be phylogenetically related to *D. cervi*. Three individuals, detected from both study sites and assigned to an undescribed *Dictyocaulus* sp., clustered with *Dictyocaulus* specimens isolated from red deer and fallow deer in previous studies. Within each of *D. cervi* and the undescribed *Dictyocaulus* sp., the newly isolated nematodes phylogenetically clustered based on their geographical origin. This study revealed the presence of *D. cervi* in Italian red deer, and an undetermined *Dictyocaulus* sp. that should be more deeply investigated. The results suggest that further analyses should be focused on population genetics of cervids and their lungworms to assess how they evolved, or co-evolved, throughout time and space and to assess the potential of transmission towards farmed animals.

**Supplementary Information:**

The online version contains supplementary material available at 10.1007/s00436-022-07773-4.

## Introduction

Parasitic nematodes of the genus *Dictyocaulus* (family Dictyocaulidae, superfamily Trichostrongyloidea) are known to affect domestic and wild ruminants (Ács et al. 2016; Höglund et al. 2003). The adult lungworms are found in the small and large airways of the host, potentially causing parasitic, often fatal, bronchitis (dictyocaulosis), especially in cattle, sheep, and farmed red deer *Cervus elaphus*. This condition, in turn, can lead to potential economic losses in livestock production (Jackson 2008; Pyziel et al. 2017).

In free-living cervids, the disease should be considered noteworthy for wildlife management with respect to the interaction with livestock (Pyziel et al. 2015), as well as to potential threats to biodiversity, wildlife conservation, and the development of the game industry (Pyziel et al. 2017; Pyziel 2018). Some species of the genus *Dictyocaulus* show host-specificity, as in the case of *Dictyocaulus viviparus* Bloch, 1782 (cattle) and *Dictyocaulus filaria* Rudolphi, 1809 (sheep and goat) (Bangoura et al. 2020). On the other side, *Dictyocaulus eckerti* Skrjabin, 1931 was until recently considered a complex of species associated with a range of cervid species (Divina et al. 2000; Pyziel et al. 2017). The *Dictyocaulus eckerti* complex was then split following the description of additional cervid-specific species, such as *Dictyocaulus capreolus* Gibbons & Höglund, 2002 in roe deer *Capreolus capreolus*, moose *Alces alces* (Gibbons and Höglund 2002) and *Dictyocaulus cervi* Pyziel 2017 in red deer (Pyziel et al. 2017). However, the host range and epidemiology of *Dictyocaulus* spp. in cervids still need to be elucidated. In fact, a higher morphological variability than currently recognized may occur in the cervid-related lungworm species (Bangoura et al. 2020), especially from the perspective of possible cross-transmission events between cervids and livestock.

Species identification is generally based on morphology for all *Dictyocaulus* species, although significant skilled labor is required for the exact identification. Additionally, in the light of some recently described species, as for *D. cervi*, the slight differences between populations suggest that the identification should be reinforced by other tools, for example molecular analyses (Bangoura et al. 2020).

Generally, dictyocaulosis in wildlife has been sporadically investigated in Italy, leading in turn to limited and/or often outdated data on the infection (Romano et al. 1980; Bregoli et al. 2006; Zanet et al. 2021). The morphological identification of *D. eckerti* and *D. cervi* requires a careful evaluation, since misidentification between the two could have occurred in the past. This aspect thus calls for updated knowledge of the epidemiology of these parasites in wild cervids.

The present work is aimed to investigate the presence of *Dictyocaulus* nematodes in wild red deer in two areas of the Italian Alps and characterize them from the molecular standpoint.

## Materials and methods

The study was conducted in two regions in the Italian Alps: (i) Valle d’Aosta (hereafter, VdA) (45°44′14″N, 7°19′14″E), in the western Alps; (ii) Lombardy, within the Stelvio National Park (hereafter, SNP) (46°28′0″N, 10°22′0″E), in the central-eastern Alps. In VdA, 104 red deer (a total of 47 males and 57 females; 36 calves, 60 yearlings, and 8 adults), hunted according to Italian national hunting law 157/1992, were checked for adult lungworms in the season 2017–2018 (October–January) in two hunting district control centers (Etroubles and Aymavilles). In SNP, 146 red deer (a total of 58 males and 88 females; 49 calves, 20 yearlings, and 77 adults), culled during a control program authorized by the Italian Institute for Environmental Protection and Research (Prot. 48585/T-A25-Ispra), were checked in the season 2018–2019 (December–February) at the checkpoint of the park (Bormio). Animals were classified as calves (< 1-year old), yearlings (1-year old) and adults (≥ 2-year old).

The respiratory tracts from the trachea to bronchioles of freshly hunted animals were dissected, cut open, and flushed with tap water. The nematodes were recovered directly from the respiratory tract or decanted washing water and observed under a Leica MZ95 stereomicroscope (Leica Microsystems, Wetzlar, Germany), as described in Pyziel et al. (2017). For further analyses, the recovered nematodes were preserved individually in 70% ethanol at + 4 °C.

The collected lungworms were cleared with lactophenol to perform morphological identification. Lungworms were observed and measured using a Leica DMLS light microscope (magnification from 100 × to 400 × ; Leica Microsystems, Wetzlar, Germany), and the identification was based on specific keys (Skrjabin et al. 1954; Gibbons et al. 1988; Pyziel et al. 2017; Bangoura et al. 2020) for all recovered lungworms. Parasite prevalence, mean intensity, mean abundance, and confidence limits were generated with the software package Quantitative Parasitology 3.0 (QP 3.0) (Rózsa et al. 2000). Parasite prevalence and mean intensity were compared using a Bootstrap 2-sample *t*-test and Fisher’s exact test, both available in QP 3.0.

For the molecular investigations, worms were selected from a subsample of positive red deer chosen randomly. One worm per red deer was subsequently randomly selected, for a total of 17 and 21 nematodes from VdA and SNP, respectively. A portion of ~ 1 mm from the central part of the nematode (lacking useful morphological features) was collected and stored at − 18 °C until further analyses. Raw lysates were obtained for genomic DNA extraction of single nematode portions and used as templates in PCR reactions (Romeo et al. 2021). Molecular analyses were performed on three marker gene sequences: 18S rDNA gene, nuclear internal transcribed spacer 2: *ITS2*, and *cytochrome oxidase I: coxI*. Amplifications were performed, according to Pyziel et al. (2017) using the following primers: NF50 (5′-TGAAATGGGAACGGCTCAT-3′) and BNR1 (ACCTACAGATACCTTGTTACGAC-3′) targeting the 18S rDNA region; ITS2F (5′-ACGTCTGGTTCAGGGTTGTT-3′) and BD3R (5′-TATGCTTAAGTTCAGCGGGT-3′) targeting the *ITS2* region. For the *coxI* region, the primer pair described in Bowles et al. (1992) were used: coxI_F (5’-TTTTTTGGGCATCCTGAGGTTTAT-3’) and coxI_R (5′-TAAAGAAAGAACATAATGAAAATG-3′). PCRs were carried out following the original protocol described for each primer pair. The obtained amplicons were run on 2% agarose gel; gel bands were excised and purified using the Wizard® SV Gel and PCR Clean-Up System (Promega, Madison, USA) following the manufacturer’s instructions and Sanger sequenced bidirectionally. Sequencing was performed using internal primers NF890 (CCTAAAGCGAAAGCATTTGCC) and NR1040 (CATACCCCAGGAACCGAA) (Pyziel et al. 2017) for the 18S rDNA fragment and using the primers given above for the *ITS2* and *coxI* amplicons. The obtained gene sequences were deposited in GenBank (see Supplementary Table S1). Their evolutionary distances, and with respect to *Dictyocaulus* spp. sequences present in GenBank, were estimated using MEGA X version 10.1.8 (Kumar et al. 2018) software and compared.

Concerning phylogenetic analyses, for each marker (18S rDNA, *ITS2*, and *coxI*) a representative selection of sequences of other *Dictyocaulus* spp. from previous studies was downloaded, together with outgroup sequences. Next, for 18 rDNA, sequences were aligned with the automated aligner of the ARB software package (Ludwig et al. 2004), and the alignment was manually refined to optimize base-pairing. For *ITS2* and *coxI*, sequences were aligned with MUSCLE (Edgar 2004). The optimal substitution model was then selected for each aligned marker with jModelTest (Darriba et al. 2012), and a maximum likelihood phylogeny was inferred with PhyML (Guindon and Gascuel 2003) with 100 bootstrap pseudo-replicates. Bayesian inference trees were inferred with MrBayes (Ronquist et al. 2012), employing three runs, each with one cold and three heated Markov chains Monte Carlo, iterating for 1,000,000 generations, with 25% burn-in.

## Results and discussion

Red deer from both study areas were infected with *Dictyocaulus* spp. In VdA, 23 out of 104 individuals tested positive for lungworms, with an overall prevalence of 22.1% (95% *CI* = 14.56–31.32). A significantly higher prevalence (*P* < 0.05) was observed in calves (33.3%; 95% *CI* = 17.6–48.4) than in yearlings (15%; 95% *CI* = 6–24); prevalence in adults (25%; 95% *CI* = 5–55) was not significantly different than in calves or yearlings (positive individuals were 12/36, 9/60, and 2/8 for calves, yearlings and adults, respectively). No significant difference was found between the number of infected males and females (29.8% and 15.8%, respectively). A total of 129 lungworms were collected (46 males and 83 females). The mean intensity was 5.57 (95% *CI* = 3.57–8.57), and the mean abundance was 1.21 (95% *CI* = 0.69–2.10).

In SNP, 32 out of 146 individuals tested positive for lungworms, with an overall prevalence of 21.9% (95% *CI* = 15.50–29.51). Prevalence in calves (40.8%; 95% *CI* = 27–54.6) was significantly higher (*P* < 0.05) than in adults (9.1%; 95% *CI* = 2.7–15.5), but not in yearlings (25%; 95% *CI* = 6–44) (positive individuals were 20/49, 5/20, and 7/77 for calves, yearlings, and adults, respectively). No significant difference was found between the number of infected males and females (27.6% and 18.2%, respectively). A total of 227 lungworms were collected (99 males and 128 females). The mean intensity was 7.09 (95% *CI* = 4.81–11.44), and the mean abundance was 1.55 (95% *CI* = 0.95–2.73). 

According to the morphology, all the lungworms could be ascribed to *D. cervi*. No significant differences between parasite prevalence and mean intensity in hosts from the two study areas were found. Overall, the prevalence in the two areas was lower than previously reported for other European countries. For example, recent investigations on *D. cervi* in red deer showed prevalence levels of about 44–68% in Poland (Pyziel et al. 2017; Pyziel 2018). Lungworm surveys in Sweden and Hungary recorded a putative *D. eckerti* prevalence of about 33% in red deer (Divina et al. 2002; Ács et al. 2016). In our study, parasite prevalence was higher in calves than in adults, confirming data reported in the literature (David 1997; Divina et al. 2002; Ács et al. 2016).

**Fig. 1:**
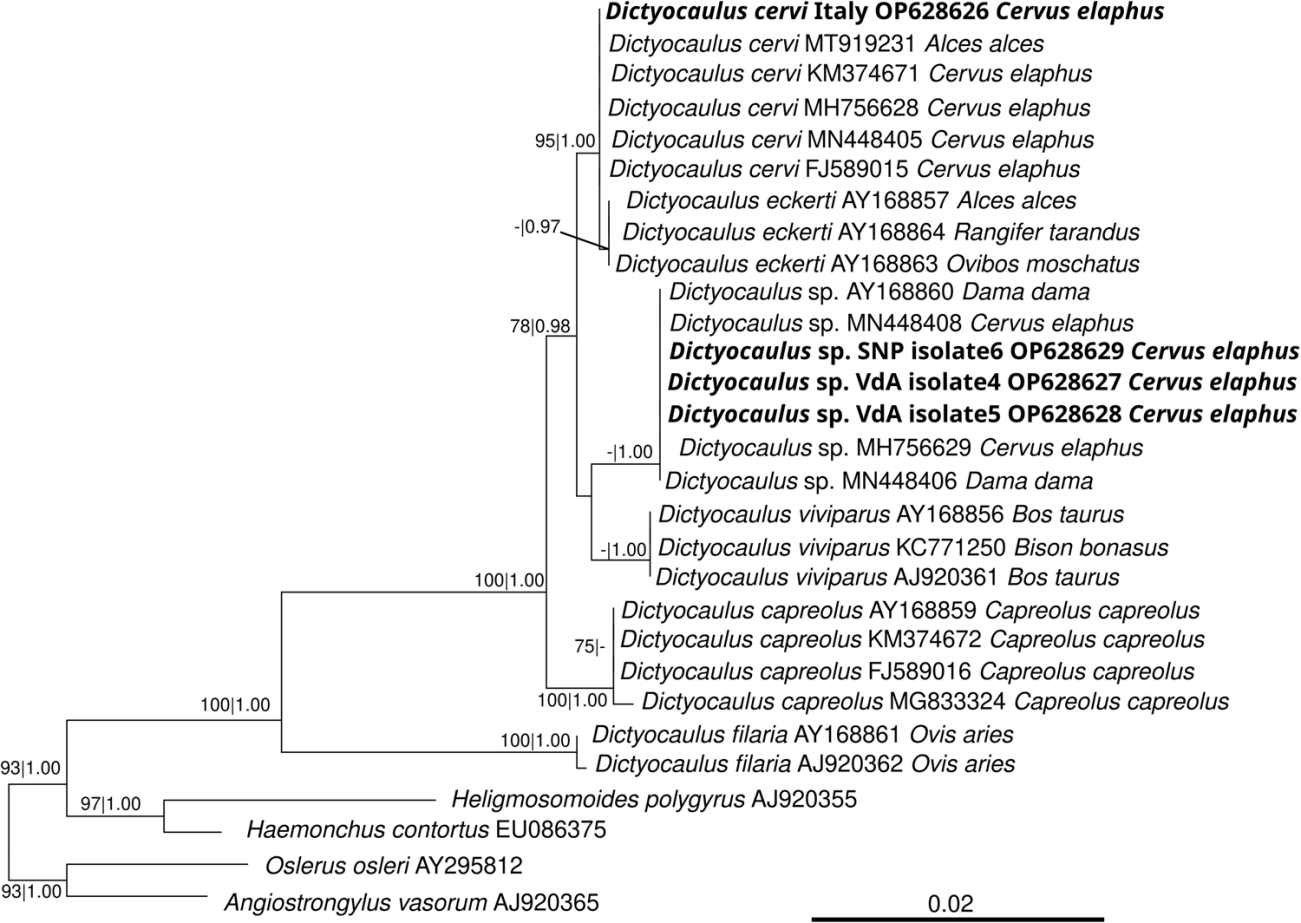
Maximum likelihood phylogenetic tree of the 18S rDNA sequences of *Dictyocaulus* spp. with the newly obtained sequences evidenced in bold. For each sequence, the NCBI accession numbers, plus the host species origin are reported. Numbers on branches report bootstrap supports with 100 pseudo-replicates and Bayesian posterior probabilities after 1.000.000 iterations. The scale bar stands for proportional sequence divergence. Support values below (70|0.85) were omitted

In the molecular analyses, all the examined lungworms tested positive for the three PCR targets (data on samples, accession numbers, ascribed species, and isolates are reported in Supplementary Table 1). The sequenced 18S rDNA and *ITS2* amplicons showed that 35 out of 38 sequences were 100% identical with sequences of *D. cervi* available in GenBank (18S rDNA — accession MH183394; *ITS2* — accession KM374673). Consistently, they were included in the *D. cervi* clade with high branch support in the respective phylogenies (Fig. 1; Supplementary Fig. 1). The 18S rDNA gene sequences of three out of 38 specimens (two from VdA and one from SNP, respectively indicated as “*Dictyocaulus* sp. VdA isolate 4,” “*Dictyocaulus* sp. VdA isolate 5”, and “*Dictyocaulus* sp. SNP isolate 6”) showed 100% identity with undescribed *Dictyocaulus* sp. found in red deer and fallow deer *Dama dama*, forming the sister group of *D. viviparus* (Fig. 1). For these three undescribed *Dictyocaulus* specimens, the obtained *ITS2* amplicons showed 93.83–100% identity with sequences belonging to *Dictyocaulus* sp. isolates present in GenBank. Contrary to the 18S rDNA gene-based phylogenetic inference, the *ITS2* gene sequences of these three *Dictyocaulus* sp. specimens from the present work did not form a sister clade with *D. viviparus* but with *D. capreolus* instead (Supplementary Fig. 1).

**Fig. 2:**
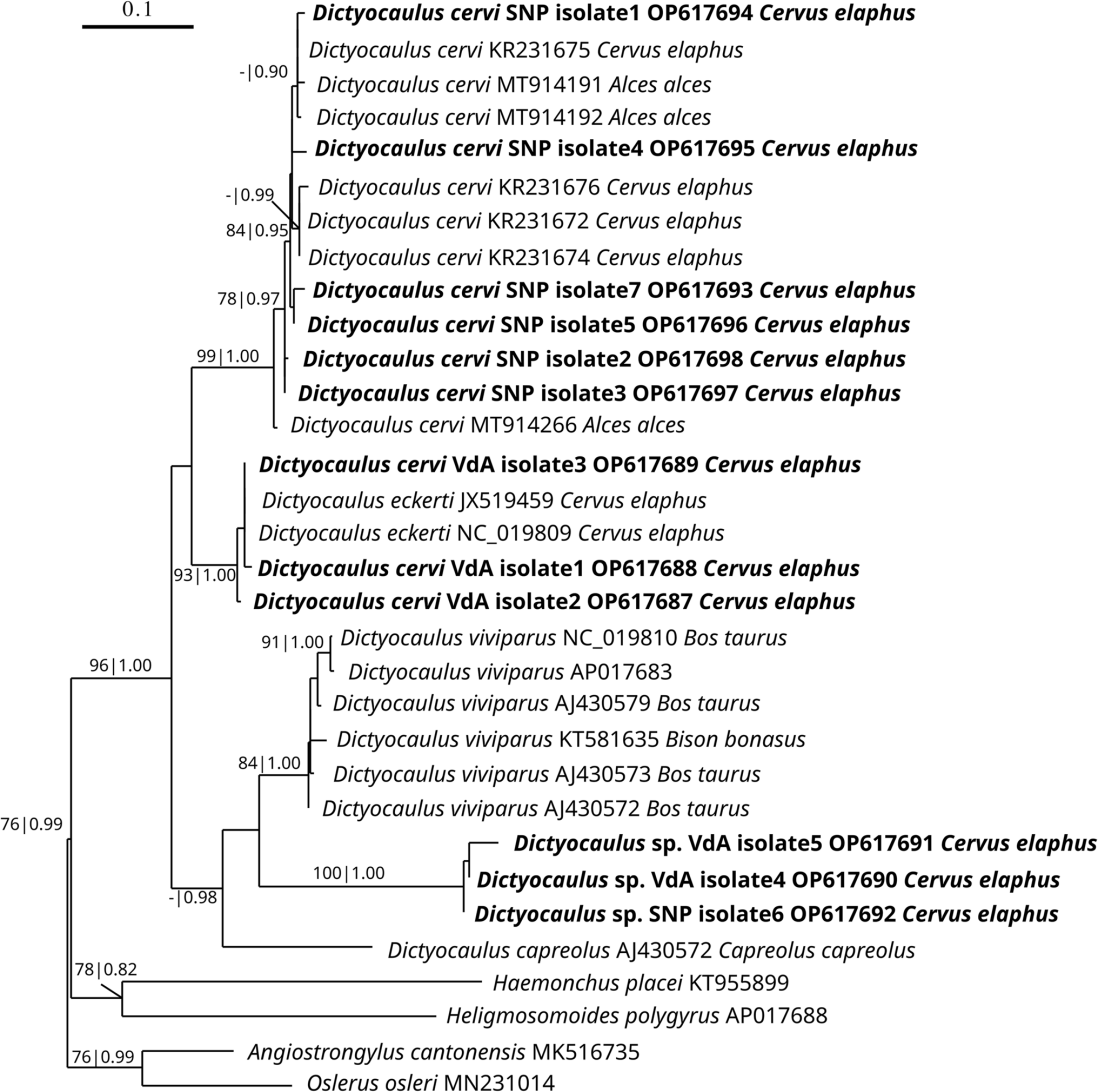
Maximum likelihood phylogenetic tree of the *coxI* sequences of *Dictyocaulus* spp. with the newly obtained sequences evidenced in bold. For each sequence, the NCBI accession numbers, plus the host species origin are reported. Numbers on branches report bootstrap supports with 100 pseudo-replicates and Bayesian posterior probabilities after 1.000.000 iterations. The scale bar stands for proportional sequence divergence. Support values below (70|0.85) were omitted

Interestingly, the phylogenetic inferences based on the mitochondrial *coxI* gene revealed population subdivisions based on the geographical area for what concerns lungworms molecularly identified as *D. cervi* using the two nuclear markers. Furthermore, *coxI* gene analysis again confirmed the presence of a separate, well-supported clade belonging to a *Dictyocaulus* sp. (Fig. 2). The *D. cervi coxI* gene sequences from SNP clustered with high support within the *D. cervi* clade (Fig. 2), together with sequences obtained from red deer and moose from central-eastern European countries (e.g., Poland and Hungary). On the contrary, sequences from VdA, previously identified as belonging to *D. cervi* based on morphology and the two nuclear markers, clustered with *D. eckerti* sequences with strong support (Fig. 2). On the one hand, a higher phylogenetic resolution within the *D. cervi/D. eckerti* clade obtained with *coxI* is not surprising. It should be considered that mitochondrial DNA is extensively employed as a marker in genetic diversity and phylogenetic analyses at various taxonomic levels for its maternal inheritance, lack of recombination, and, specifically, its fast evolutionary rate (Boore 1999; Blouin 2002; Yong et al. 2015; Zhao et al. 2022). On the other hand, the non-congruence in the species assignment of some specimens to *D. eckerti* (or *D. cervi)* is possibly only apparent, considering that *D. cervi* was only recently circumscribed from the original *D. eckerti* complex (Pyziel et al. 2017). Therefore, to address this point, a careful and comprehensive comparison of the morphological and genetic data obtained in previous studies would be required, which is far beyond the aims of the present study. Thus, we will treat those genetic variations mostly from a bio-geographical perspective. The geographical separation may associate with the genetic differentiation between lungworms in the two areas, and this is partly supported by the historical dynamics of red deer in the Italian Alps. Red deer populations in Italy faced a drastic reduction over the past centuries, nearly reaching extinction, until the half of the twentieth century when the species recolonized the southern Alps both spontaneously and through reintroductions and restocking (Mattioli et al. 2001). Recolonization first occurred spontaneously in the central-eastern Alps towards the end of the 1940s, with red deer entering from neighboring countries (Mattioli et al. 2001). This aspect may explain why SNP *coxI* gene sequences cluster with high support with *D. cervi* sequences from central-eastern Europe. In the western Alps, including Valle d’Aosta, red deer demographic recovery was possible thanks to spontaneous recolonization from the neighboring Swiss regions as well as to occasional reintroductions of subjects from the eastern Alps (Tarello 1991; Mattioli et al. 2001; Carnevali et al. 2009). This might have impacted the genetic differences observed between the lungworm populations from the two study areas. Previous studies performed on populations genetics of large lungworms in wild deer in Hungary cast the possibility of divergence between *Dictyocaulus* species linked to their host populations (Àcs et al. 2016). Sequence differences between the two nematode populations from VdA and SNP are about 8.7% (Supplementary Table 2), which is lower than the empirical 10% threshold applied to species differentiation in nematodes (Ács et al. 2016; Blouin 2002). This is in accordance with sequence variation observed in other studies (Pyziel 2018). No *coxI* gene sequences of *D. cervi/D. eckerti* from central-western European areas (e.g., France, Switzerland, and Germany) are currently available, thus limiting the geographical resolution of phylogenetic inferences of nematodes belonging to this complex.

The three novel sequences forming a separate clade from the *D. cervi*/*D. eckerti* complex showed nucleotide differences of the *coxI* gene > 12% with respect to *D. cervi*/*D. eckerti*. Geographical clustering was also observed in the *coxI* gene for this putative undescribed *Dictyocaulus* species, with the two VdA isolates more closely related to each other than the SNP one, confirming the clustering results obtained with the *ITS2* gene (Supplementary Fig. 1). In the *coxI* analysis, the undescribed *Dictyocaulus* sp. was defined as a sister group of *D. viviparus*, supporting the result obtained through the 18S rDNA phylogenetic inference. Interestingly, a previous study reported *coxI* sequences from an undescribed *Dictyocaulus* sp. clustering as a sister group of *D. viviparus* as well (namely *Dictyocaulus* sp. S-HU in Hungary; Ács et al. 2016). However, a direct comparison is currently not possible since, in the present study, a neighboring gene region of the cox*I* gene was analyzed, compared to those of *Dictyocaulus* sp. S-HU (Ács et al. 2016). In any case, regarding the phylogenetic positioning within the genus of the *Dictyocaulus* sp. from this study, the three markers employed provided neither fully concordant nor fully supported reconstructions (Figs. 1 and 2; Supplementary Fig. 1). Such differences may be explained by insufficient phylogenetic resolution (e.g., available *ITS2* sequences, which provided the alternative reconstruction, compared to the other two markers, were, on average, relatively short and not fully reciprocally overlapping due to the usage of different primers among studies). Thus, these results should be considered preliminary and taken with some caution. Nevertheless, all three markers consistently indicated that the three *Dictyocaulus* specimens likely belonging to a separate, still undescribed, species.

In summary, the present results provide the first detection of *D. cervi* in red deer in Italy, representing a jigsaw piece for the epidemiological overview of dictyocaulosis in European wildlife. Our study underlines the importance of molecular analyses for lungworm identification, specifically for *Dictyocaulus* species in cervids. The presence of the *Dictyocaulus* sp. in red deer indicates that further efforts are needed to investigate and define the host range of this still undescribed species to evaluate its potential ability to reach domestic livestock. Future studies should investigate the taxonomy, phylogeny, ecology, and epidemiology of *Dictyocaulus* spp. at large spatial scale, as this would provide essential indications for both wildlife and lungworm management.

## Supplementary Information

Below is the link to the electronic supplementary material.

Supplementary file1 (PNG 880 KB)

Supplementary file2 (XLSX 14 KB)

Supplementary file3 (DOCX 13 KB)

## Data Availability

The obtained sequences are deposited in GenBank under the accession numbers OP617687-OP617698 and OP628626-OP628632.
