# Supplementary figures and images for: Molecular characterization of Dictyocaulus nematodes in wild red deer Cervus elaphus in two areas of the Italian Alps
Source: Parasitol Res. 2023 Jan 14;122(3):881–7. doi: 10.1007/s00436-022-07773-4 (PMC9988769; doi:10.1007/s00436-022-07773-4)

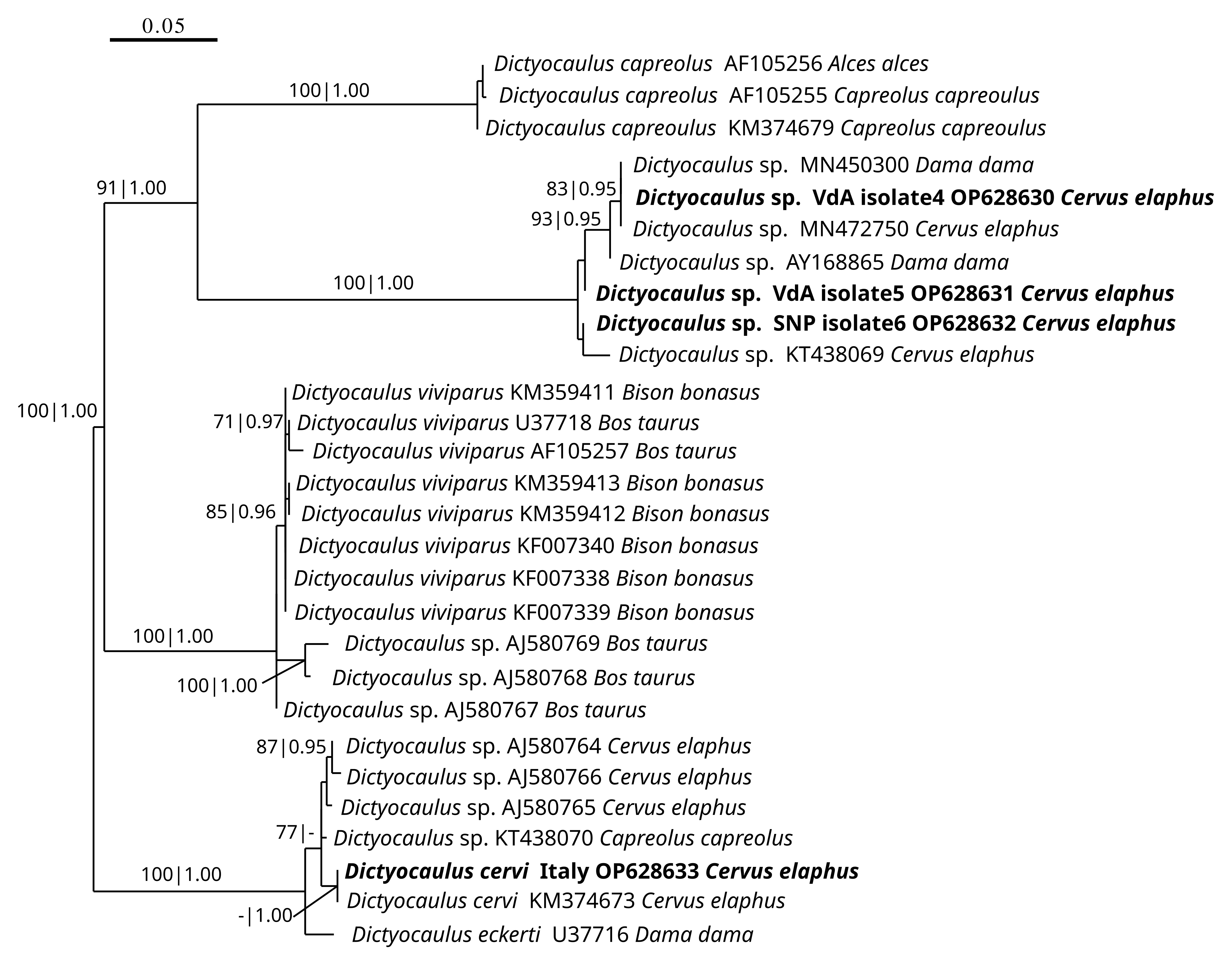

Supplement: Supplementary file 1 — Supplementary file1 (PNG 880 KB) [file 436_2022_7773_MOESM1_ESM.png]
