# Supplementary material for: Molecular characterization of Dictyocaulus nematodes in wild red deer Cervus elaphus in two areas of the Italian Alps
Source: Parasitol Res. 2023 Jan 14;122(3):881–7. doi: 10.1007/s00436-022-07773-4 (PMC9988769; doi:10.1007/s00436-022-07773-4)
Supplement: Supplementary file 3 — Supplementary file3 (DOCX 13 KB) [file 436_2022_7773_MOESM3_ESM.docx]

**Supplementary Table 2**

|  | *D. cervi* VdA | Dictyocaulus sp | *D. cervi* SNP |
| --- | --- | --- | --- |
| *D. cervi* VdA |  |  |  |
| *Dictyocaulus* sp | 0.1279 |  |  |
| *D. cervi* SNP | 0.0866 | 0.1595 |  |

Estimates of Evolutionary Divergence over Sequence Pairs between Groups for the *cytochrome oxidase I* (*coxI*) gene. The number of base substitutions per site from averaging over all sequence pairs between groups are shown. Analyses were conducted using the Maximum Composite Likelihood model (Tamura et al., 2004). This analysis involved 12 nucleotide sequences. All ambiguous positions were removed for each sequence pair (pairwise deletion option). There were a total of 328 positions in the final dataset. Evolutionary analyses were conducted in MEGA X (Kumar et al., 2018).

SNP = Stelvio National Park study site; VdA = Valle d’Aosta study site.

Tamura K., Nei M., and Kumar S. (2004). Prospects for inferring very large phylogenies by using the neighbor-joining method. Proceedings of the National Academy of Sciences (USA) 101:11030-11035.

Kumar S., Stecher G., Li M., Knyaz C., and Tamura K. (2018). MEGA X: Molecular Evolutionary Genetics Analysis across computing platforms. Molecular Biology and Evolution 35:1547-1549.
